# Expression of membrane cofactor protein (MCP, CD46) in human liver diseases

**DOI:** 10.1038/sj.bjc.6690604

**Published:** 1999-08

**Authors:** N Kinugasa, T Higashi, K Nouso, H Nakatsukasa, Y Kobayashi, M Ishizaki, N Toshikuni, K Yoshida, S Uematsu, T Tsuji

**Affiliations:** The First Department of Internal Medicine, Okayama University Medical School, 2-5-1 Shikata-cho, Okayama-city, Okayama, 700-8558, Japan

**Keywords:** membrane cofactor protein, hepatocellular carcinoma, liver, complement

## Abstract

Membrane cofactor protein (MCP, CD46) is one of the complement regulatory proteins, and is widely distributed in human organs and protects cells from complement-mediated cytotoxicity. We analysed the distribution and the intensities of MCP in liver diseases and evaluated the role of MCP during hepatocarcinogenesis. Western blot analysis revealed that relative densities (density of the sample/density of the standard sample) of MCP in 27 HCC, 18 liver cirrhosis, nine chronic hepatitis and 12 normal liver were 0.63 ± 0.23, 0.21 ± 0.07, 0.25 ± 0.10 and 0.11 ± 0.03 (mean ± s.d.) respectively. MCP expression in hepatocellular carcinoma (HCC) was significantly higher than that in both liver cirrhosis and chronic hepatitis (*P* < 0.01). The difference in the tumour sizes, the grades of differentiation and viral marker status did not affect the expression. Immunohistological analysis revealed that MCP was distributed mainly in the basolateral membrane of the hepatic cord in non-cancerous liver, along with endothelial cells and bile duct cells. In HCC, the protein was observed on the membrane in a non-polarized fashion. These data suggest that HCC cells acquire the increased MCP expression in a development of HCC and may escape from tumour-specific complement-mediated cytotoxicity. © 1999 Cancer Research Campaign


British Journal of Cancer (1999) 80(11), 1820–1825
© 1999 Cancer Research Campaign
Article no. bjoc.1999.0604

### Expression of membrane cofactor protein (MCP, CD46) in human liver diseases

**N Kinugasa, T Higashi, K Nouso, H Nakatsukasa, Y Kobayashi, M Ishizaki, N Toshikuni, K Yoshida, S Uematsu and T Tsuji**

The First Department of Internal Medicine, Okayama University Medical School, 2-5-1 Shikata-cho, Okayama-city, Okayama 700-8558 Japan

**Summary** Membrane cofactor protein (MCP, CD46) is one of the complement regulatory proteins, and is widely distributed in human organs and protects cells from complement-mediated cytotoxicity. We analysed the distribution and the intensities of MCP in liver diseases and evaluated the role of MCP during hepatocarcinogenesis. Western blot analysis revealed that relative densities (density of the sample/density of the standard sample) of MCP in 27 HCC, 18 liver cirrhosis, nine chronic hepatitis and 12 normal liver were 0.63 ± 0.23, 0.21 ± 0.07, 0.25 ± 0.10 and 0.11 ± 0.03 (mean ± s.d.) respectively. MCP expression in hepatocellular carcinoma (HCC) was significantly higher than that in both liver cirrhosis and chronic hepatitis ($P < 0.01$). The difference in the tumour sizes, the grades of differentiation and viral marker status did not affect the expression. Immunohistological analysis revealed that MCP was distributed mainly in the basolateral membrane of the hepatic cord in non-cancerous liver, along with endothelial cells and bile duct cells. In HCC, the protein was observed on the membrane in a non-polarized fashion. These data suggest that HCC cells acquire the increased MCP expression in a development of HCC and may escape from tumour-specific complement-mediated cytotoxicity.

**Keywords:** membrane cofactor protein; hepatocellular carcinoma; liver; complement

The human complement system is one of the major effectors of immune response and is involved in many cytolytic activities. Autologous human cells, but not foreign cells, are usually protected from this complement-mediated cytolysis by expressing complement regulatory proteins (CRP) on their membranes (Hourcade et al, 1989). The main CRP currently identified is membrane cofactor protein (MCP, CD46), decay-accelerating factor (CD55), Protection (CD59) and complement receptor 1 (CR1) (Davis et al, 1989; Hourcade et al, 1989; Lublin and Atkinson, 1989; Lachmann, 1991; Liszewski et al, 1991).

MCP is composed of repeating units of approximately 60 amino acids known as short consensus repeats (SCRs), a serine–threonine–proline-rich region (STP) containing several *O*-linked glycosylation sites, a transmembrane region, a basic amino acid anchor and a cytoplasmic tail (Davis et al, 1989; Theodore et al, 1991; Matsumoto et al, 1992). This CRP is widely distributed and is expressed in fibroblast, endothelial cells and epithelial cells in many organs including liver (McNearney et al, 1989; Liszewski et al, 1991; Johnstone et al, 1993).

MCP serves as a cofactor for the plasma serine protease factor I-mediated cleavage of C3b/C4b, the activated component of complement cascade, and down-regulates the critical step of C3 activation. C3b, the activated form of C3, is induced via both the classical pathway and an alternative pathway and activates the terminal lytic complement sequence including the formation of the membrane attack complex (Seya and Atkinson, 1989; Seya et al, 1994). Therefore, the down-regulation of C3b by MCP is crucial for preventing autologous cells from complement-dependent cytotoxicity.

In recent reports, increased expression of MCP was observed in cells of human breast, stomach, colon, ovary, cervix and non-small-cell lung cancer (Hofman et al, 1994; Inoue et al, 1994; Niehans et al, 1996; Bjørge et al, 1997; Simpson et al, 1997). Expression of MCP by tumours raises the possibility that the complement system participates in host immune responses to cancers. There are several in vitro experiments that support this hypothesis; the number of CD55 molecules per cancer cell was reported to correlate with the degree of complement resistance, and the expression of MCP in CD55-negative cell lines protected cells from complement-mediated lysis (Cheung et al, 1988; Seya et al, 1990).

Liver produces complement components and expresses MCP under normal conditions (Nagura et al, 1985; Scoazec et al, 1994), but little is known about MCP expression in pathological conditions of the liver, including human hepatocellular carcinoma (HCC). In order to analyse the role of MCP in the development of HCC, we examined the expression of MCP in various grades of HCC as well as in non-cancerous liver tissue.

### MATERIALS AND METHODS

### Samples

Twenty-seven HCC samples (23 surgically resected, four autopsied), 27 corresponding adjacent non-cancerous livers (18 liver cirrhosis, nine chronic hepatitis) and 12 normal livers were studied. Morphologically and serologically normal liver samples were obtained from autopsies of patients who died of cardiac failure or lung diseases. Histological grades of HCC (eight well-differentiated, 19 moderately differentiated) were determined

*Received 16 September 1998*
*Revised 28 January 1999*
*Accepted 4 February 1999*

*Correspondence to:* T Higashi

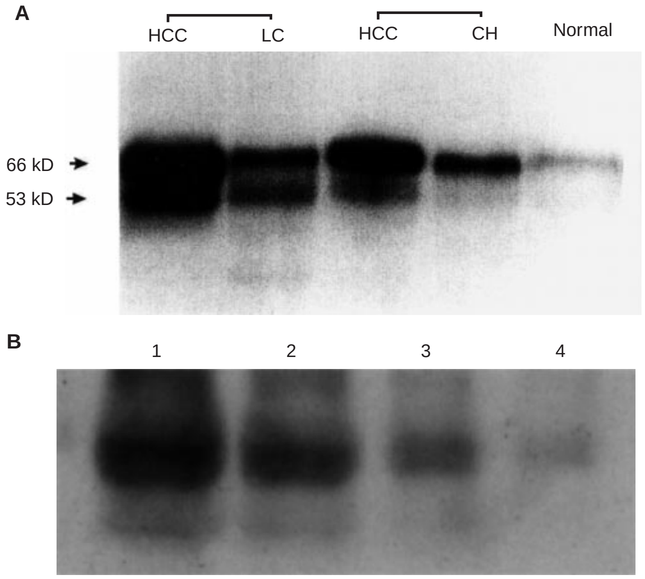

**Figure 1** Western blot analysis of MCP. MCP expression in HCC (66 kDz, 53 kDz) was significantly higher than that in both liver cirrhosis and chronic hepatitis. The normal liver showed the lowest degree of expression among the groups examined (**A**). The band intensity was correlated with the amount of a standard sample applied (**B**). The amount of protein and the corresponding relative density were as follows: lane 1, 20 µg, 1.00; lane 2, 10 µg, 0.56; lane 3, 5 µg, 0.28; and lane 4, 2.5 µg, 0.06. HCC = hepatocellular carcinoma, LC = liver cirrhosis, CH = chronic hepatitis, Normal = normal liver

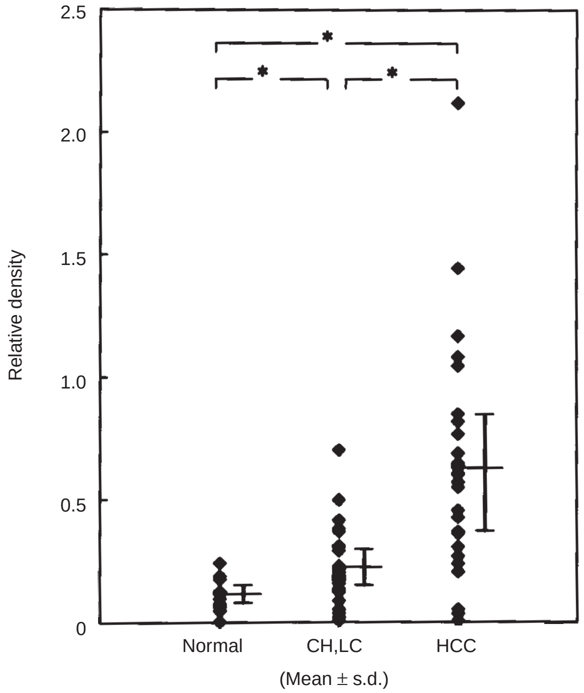

**Figure 2** MCP expression in liver diseases. Significant differences were observed between each group. Relative density = density of the sample/density of the standard sample. HCC = hepatocellular carcinoma, LC = liver cirrhosis, CH = chronic hepatitis, Normal = normal liver, *$P$<0.01

according to the criteria outlined by Liver Cancer Study Group of Japan (Liver Cancer Study Group of Japan, 1989). Of the 27 patients, nine (33%) were women, and patients' ages ranged from 25 to 78 years (mean = 62.0 years). Six (22%) were positive for hepatitis B virus surface antigen (HBs-Ag) and 14 (52%) were positive for hepatitis C virus antibody (HCV-Ab). Informed consent was obtained from all the patients for the experimental use of the samples.

### Tissue preparation

The samples were immediately frozen with dry ice after surgery or autopsy and stored at –80°C until use. A part of each sample was fixed with periodate–lysine–paraformaldehyde and embedded in OCT compound (Lab Tek Products, Naperville, IL, USA) for immunohistochemistry.

### Western blot analysis

Frozen liver tissues were homogenized in 8 mM 3-[(3-cholamido-propyl)-dimethylammonio]-1-propanesulphate in Tris-buffered saline supplemented with 1 mM phenylmethyl sulphonyl fluoride and allowed to solubilize for 30 min at 20°C. After incubation, insoluble cell debris was removed by centrifugation at 100 000 ***g*** for 30 min as previously reported (Simpson et al, 1993). Protein concentration of the tissue extracts was determined using the method of Bradford (1976). An equal amount of the protein (20 µg) was separated by 12.5% sodium dodecyl sulphate-poly-acrylamide gel electrophoresis under a non-reducing condition and then transferred to a nitrocellulose membrane. The membrane was blocked with 5% non-fat dry milk in phosphate-buffered saline (PBS) containing 0.1% Tween-20, and incubated with mouse monoclonal antibody against human MCP (J4-48, Immunotech, Marseille-Luminy, France) diluted 1:1000 with PBS for 1 h at room temperature. The blots were washed in PBS– Tween-20 and then incubated with horseradish peroxidase-labelled anti-mouse immunoglobulin (Amersham Japan, Tokyo, Japan) for 1 h at room temperature. After washing with PBS– Tween-20, the membrane was reacted with chemiluminescence solution (ECL Western blotting detection system, Amersham Japan, Tokyo, Japan), and the signal was visualized on autoradiography film. We used one of the moderately differentiated HCC as the standard sample, which showed the highest band intensity in a preliminary Western blot study. The intensity of the signal was measured using a densitometer (Molecular Dynamics Scanning Imager 300SX) and was defined as $\Sigma(ODi)$, where ODi is the densitometer output (arbitrary units) above background at position i. The result was expressed as relative density (density of the sample/density of the standard sample). All experiments were performed in duplicate.

### Immunohistochemistry

Six moderately differentiated HCC samples, corresponding non-cancerous liver tissues and one normal liver tissue were examined immunohistochemically. Tissue sections (4 µm) were cut in a cryostat, placed on sialynized slides (DAKO, Tokyo, Japan) and allowed to air-dry for 30 min. After rehydration in PBS, endogenous peroxidase was destroyed by 3% hydrogen peroxide treatment for 30 min and non-specific reactivity was blocked by 10-min incubation at room temperature with 10% normal rabbit serum. The sections were reacted with monoclonal antibody against human MCP (J4-48) diluted 1:1000 with PBS for 1 h at

© 1999 Cancer Research Campaign

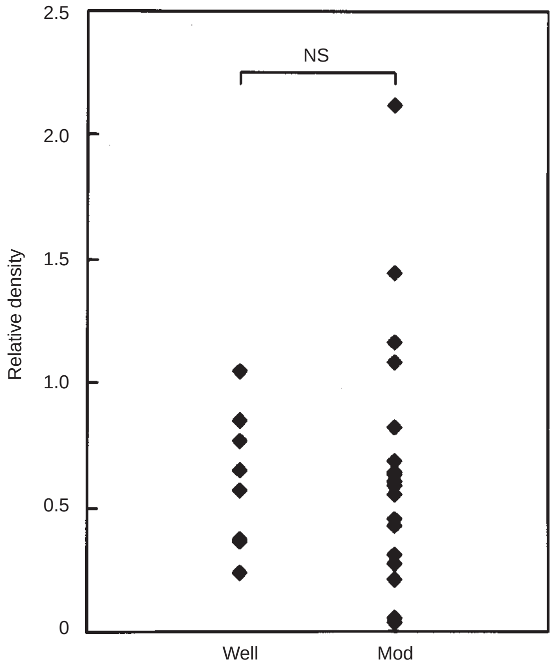

Figure 3 MCP expression in different grades of HCC. Well = well-differentiated HCC, Mod = moderately differentiated HCC, NS = not significant

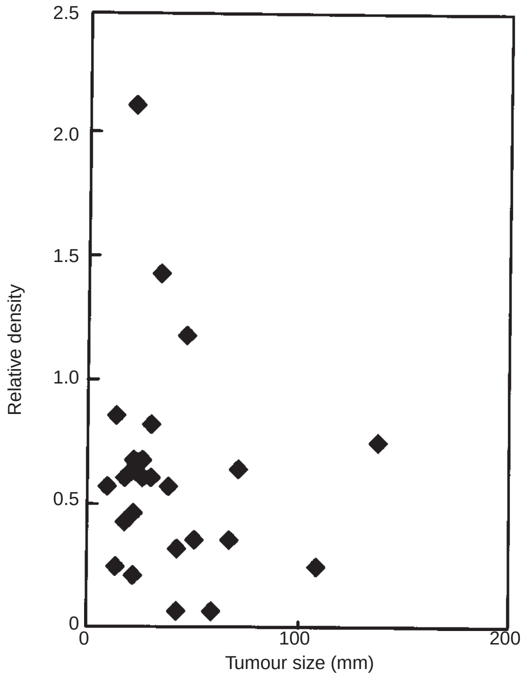

Figure 5 MCP expression in different size of HCCs. No correlation between MCP expression and the size of HCC was observed ($r = 0.15$)

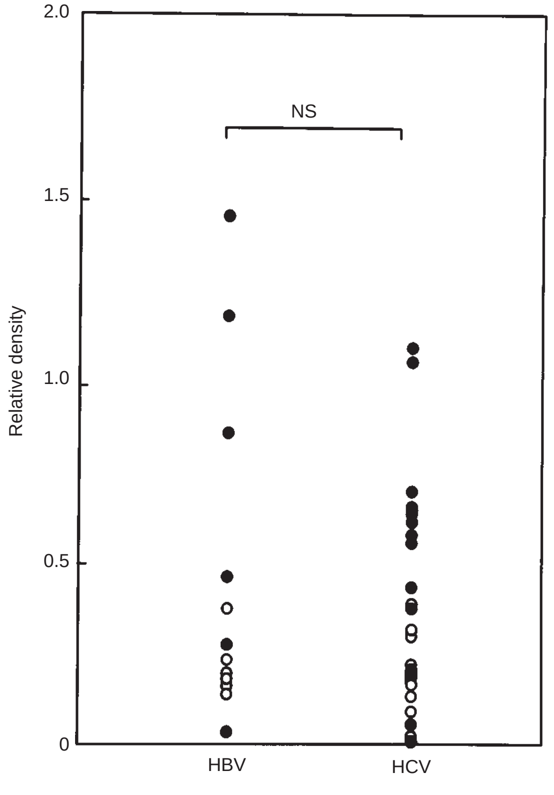

Figure 4 MCP expression and hepatitis virus expression. HBV = HBs-Ag positive, HCV = HCV-Ab positive. (○) Chronic hepatitis and liver cirrhosis, (●) hepatocellular carcinoma, NS = not significant room temperature. Biotin-conjugated anti-mouse immunoglobulin was used as the second antibody followed by a treatment with peroxidase–streptavidin complex (Histofine, Nichirei, Japan). The sections were stained with 30% 3,3′-diaminobenzidine tetrahydrochloride (Histofine, Nichirei, Japan) and methyl green was used for nuclear counterstaining. In each experiment, three controls were always prepared: omission of the primary antibody, incubation with an isotypic antibody, and incubation with normal mouse serum.

### Statistics

Statistical significance was evaluated by means of $\chi^2$ test and correlation between the size of HCC and MCP expression was analysed by linear regression.

### RESULTS

One or two MCP isoforms were expressed in each sample of liver tissue. The molecular weights of the isoforms were approximately 53 kDa and 66 kDa, although subtle size differences existed between each sample (Figure 1A). Individual variation of the ratio of 66 kDa to 53 kDa was observed; however, the 66 kDa isoform were predominant in all samples examined. The densitometric data was well correlated with the amount of samples applied (Figure 1B). Relative density of total MCP components in HCC, liver cirrhosis, chronic hepatitis and normal liver was 0.63 ± 0.23, 0.21 ± 0.07, 0.25 ± 0.10 and 0.11 ± 0.03 respectively (Figure 2). MCP expression in HCC was significantly higher than that in both liver cirrhosis and chronic hepatitis ($P < 0.01$). The normal liver showed the lowest expression among the groups examined

 © 1999 Cancer Research Campaign

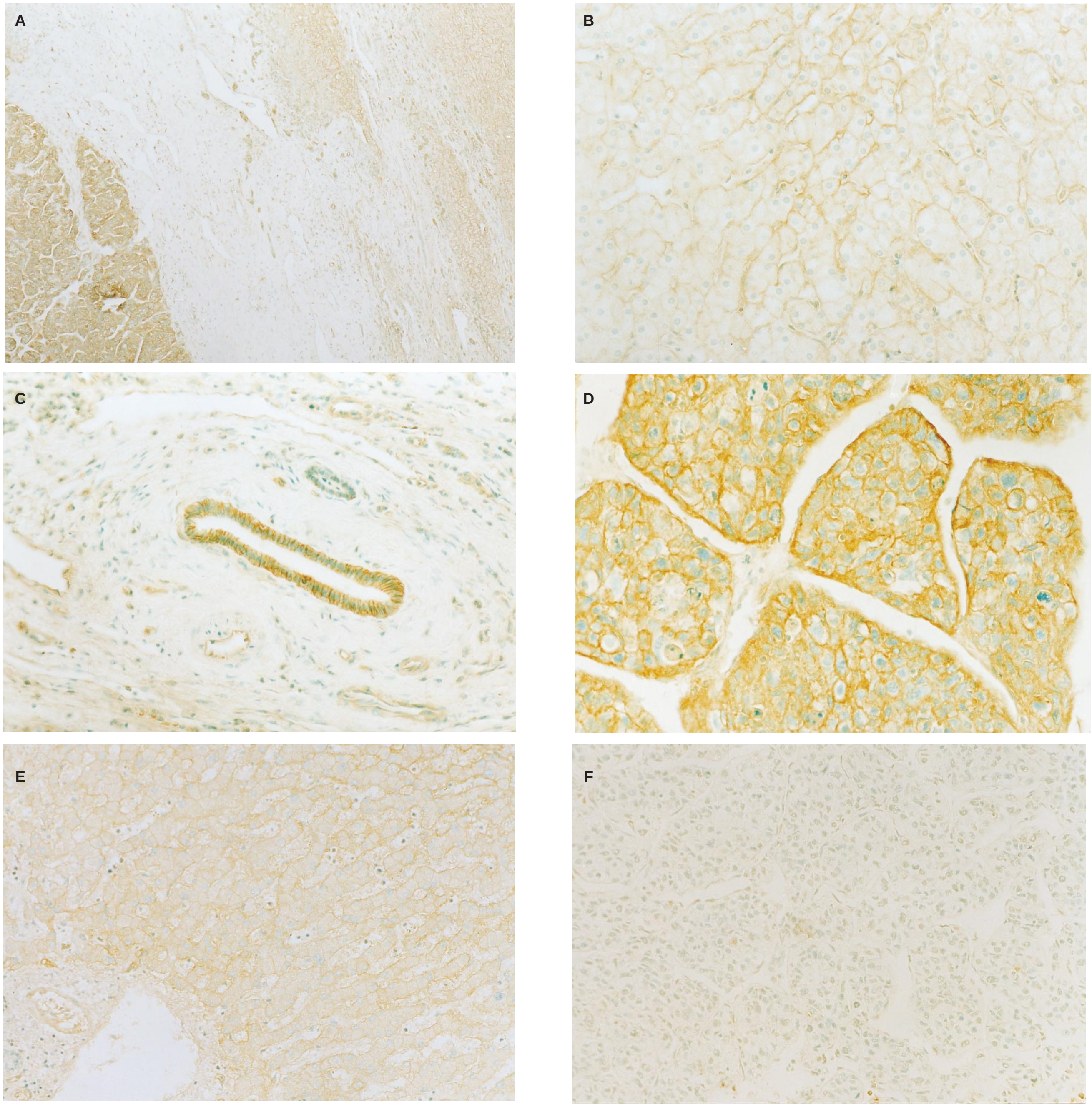

**Figure 6** Immunostaining of MCP. Note that the expression in HCC (left lower side) was higher than that of liver cirrhosis (right upper side, ×100) (**A**). MCP was expressed on the basolateral surface of hepatic cords in liver cirrhosis (×200) (**B**). Vascular endothelial cells and bile duct cells were stained with MCP antibody (×200) (**C**). Strong expression of MCP was observed at the cell membrane of moderately differentiated hepatocellular carcinoma in non-polarized fashion (×400) (**D**). Normal liver (×200) (**E**). The consecutive section of (**D**) was stained with normal mouse serum as a negative control (×200) (**F**). Methyl green was used for nuclear counterstaining

($P < 0.01$). There was no significant difference in the expression of MCP among different histological grades of HCC or the status of hepatitis virus infection (Figures 3 and 4). The size of HCC did not correlate with MCP expression ($r = 0.15$, Figure 5).

MCP expression was immunohistochemically detected in all six HCC and corresponding non-cancerous livers (Figure 6A). MCP was homogeneously distributed on the cellular membrane of the hepatocyte in non-cancerous sections. The expression was stronger on the basolateral membrane of the hepatic cords than on the intercellular surface (Figure 6B). MCP was also detected in sinusoidal endothelial cells, vascular endothelial cells, and a strong expression was observed in bile duct cells (Figure 6C). In HCC, the expression was stronger than in the non-cancerous tissue in all cases examined. The main locus of the protein was the cell membrane as was similarly observed in non-cancerous lesion. MCP was observed in a non-polarized fashion in each cell, and the cellular distribution of MCP was homogeneous in each trabeculum of HCC (Figure 6D). MCP was expressed on the cellular

*© 1999 Cancer Research Campaign*

membrane of the hepatocyte in normal liver as was observed in non-cancerous tissue (Figure 6E). All controls were consistently negative for staining (Figure 6F).

### DISCUSSION

In this study, we first demonstrated that MCP is up-regulated in chronically injured livers including HCC. CRP is necessary for cells physiologically exposed to blood, such as erythrocytes and vascular endothelial cells, to protect themselves against the complement-dependent cytotoxicity. In contrast to the other epithelial cells, hepatocytes are in intimate contact with blood because of the fenestrated sinusoidal endothelium and the lack of an organized basement membrane (Seya et al, 1990). Furthermore, hepatocytes produce complement, and their sinusoidal membranes are positive for C3 and C4 (Nagura et al, 1985). Recurrent necrosis and regeneration of the hepatocyte that occur in chronic liver diseases cause damage to liver function and lead to accumulation of endotoxin, xenobiotics and immune complexes. Under these conditions, hepatocytes may need to protect themselves from the attack by complement by virtue of the increased expression of MCP.

It has been recently reported that the increased expression of CRPs, such as MCP, CD59, was observed in many human cancers and carcinoma cell lines (Hakulinen and Meri, 1994; Hofman et al, 1994; Inoue et al, 1994; Mäenpää et al, 1996; Gorter et al, 1996; Niehans et al, 1996; Bjørge et al, 1997; Simpson et al, 1997). The expression of CD55 was also enhanced in the lumen of the colorectal cancer glands (Inoue et al, 1994). In the present study, the expression of the MCP in HCC was significantly higher than in that of normal liver, chronic hepatitis and liver cirrhosis. CD55 and CD59 are two other major CRPs; however, our preliminary study demonstrated that CD55 was not expressed in liver parenchymal cells including HCC, and the enhancement of CD59 was not observed in most of the HCC (data not shown). Therefore, it seems that MCP plays a major role in inhibiting the complement activation in HCC.

The increase of MCP expression is an early event in the progression of HCC, because the expression is enhanced in HCC regardless of the size and the state of differentiation. There are several possible reasons for MCP expression in HCC being much higher than that in chronic hepatitis or liver cirrhosis. First, $CH_{50}$ in patients with HCC is higher than that in patients without HCC (Matsumura et al, 1981). Hence, HCC cells that express high amounts of MCP may be selected by immunological pressure during tumour development. Second, MCP expression is known to be up-regulated in human fetal liver (Simpson et al, 1993). Therefore, MCP may be expressed with dedifferentiation of hepatocytes during hepatocarcinogenesis.

Loss of polarity of the cells is frequently observed in many neoplasms. However, the mechanism of the loss is not clear. The cytoplasmic tail is the key to maintaining basolateral polarization of MCP molecule. Isoforms containing cytoplasmic tail 1 are transported to the cell surface more rapidly than their tail 2 counterparts and the deletion of the cytoplasmic tail abolishes this polarized transport (Maisner et al, 1996). Our present immunohistochemical observation, in which HCC tended to lose the polarization of MCP, may be caused by the deletion or mutation of the cytoplasmic tail in HCC, although we could not detect clear size differences of MCP by Western blotting analysis.

Further analysis is needed of MCP expression, differences with the physiological roles of each isoform, and the polarization signal of MCP in HCC. Eventually, the selective reduction of MCP expression on the cell surface of cancer cells by antisense or ribozyme may be an effective therapeutic strategy against HCC.

### ACKNOWLEDGEMENTS

We thank the First Department of Surgery, Okayama University Medical School and Drs. Yoshiyuki Shimamura (Chiba-Nishi Hospital, Japan), Tadakazu Matsuda (Matsuda Hospital, Japan), and Kazuhiko Morii (Himeji Red Cross Hospital) for providing a portion of the human liver samples. This work was supported by a grant from the Intractable Liver Diseases Research Committee, the Japanese Ministry of Health and Welfare.

### REFERENCES

Bradford MM (1976) A rapid and sensitive method for the quantitation of microgram quantities of protein using the principle of protein-dye binding. *Anal Biochem* **72**: 248

Bjørge L, Hakulinen J, Wahlström T, Matre R and Meri S (1997) Complement-regulatory proteins in ovarian malignancies. *Int J Cancer* **70**: 14–25

Cheung N-KV, Walter EI, Smith-Mensah WH, Tatnoff WD, Tykocinski ML and Medof ME (1988) Decay-accelerating factor protects human tumor cells from complement-mediated cytotoxicity in vitro. *J Clin Invest* **81**: 1122–1128

Davies A, Simmons DL, Hale G, Harrison RA, Tighe H, Lachmann PJ and Waldmann H (1989) CD59, an LY-6 like protein expressed in human lymphoid cells, regulates the action of the complement membrane attack complex on homologous cells. *J Exp Med* **170**: 637–645

Gorter A, Blok VT, Haasnoot WHB, Ensink NG, Daha MR and Fleuren GJ (1996) Expression of CD46, CD55, and CD59 on renal tumor cell lines and their role in preventing complement-mediated tumor cell lysis. *Lab Invest* **74**: 1039–1049

Hakulinen J and Meri S (1994) Expression and function of the complement membrane attack complex inhibitor protectin (CD59) on human breast cancer cells. *Lab Invest* **71**: 820–827

Hofman P, Hsi B-L, Manie S, Fenichel P, Thyss A and Rossi B (1994) High expression of the antigen recognized by the monoclonal antibody GB24 on human breast carcinomas: a preventive mechanism of malignant tumor cells against complement attack? *Breast Cancer Res Treat* **32**: 213–219

Hourcade D, Kolers VM and Atkinson JP (1989) The regulators of complement activation (RCA) gene cluster. *Adv Immunol* **45**: 381–416

Inoue H, Mizuno M, Uesu T, Ueki T and Tsuji T (1994) Distribution of complement regulatory proteins, decay-accelerating factor, CD59/homologous restriction factor 20 and membrane cofactor protein in human colorectal adenoma and cancer. *Acta Med Okayama* **48**: 271–277

Johnstone RW, Loveland BE and Mckenzie IFC (1993) Identification and quantification of complement regulator CD46 on normal human tissues. *Immunology* **79**: 341–347

Lachmann PJ (1991) The control of homologous lysis. *Immunol Today* **12**: 312–315

Liszewski MK, Post TW and Atkinson JP (1991) Membrane cofactor protein (MCP or CD46): newest member of the regulators of complement activation gene cluster. *Annu Rev Immunol* **9**: 431–460

Liver Cancer Study Group of Japan (1989) The general rules for the clinical and pathological study of primary liver cancer. *Jpn J Surg* **19**: 98–129

Lublin DM and Atkinson JP (1989) Decay-accelerating factor: biochemistry, molecular biology and function. *Annu Rev Immunol* **7**: 35–58

Mäenpää A, Junnikkala S, Hakulinen J, Timonen T and Meri S (1996) Expression of complement membrane regulators membrane cofactor protein (CD46), decay accelerating factor (CD55), and protectin (CD59) in human malignant gliomas. *Am J Pathol* **148**: 1139–1152

Maisner A, Liszewski MK, Atkinson JP, Schwartz-Albiez R and Herrler G (1996) Two different cytoplasmic tails direct isoforms of the membrane cofactor protein (CD46) to the basolateral surface of Madin-Darby canine kidney cells. *J Biol Chem* **271**: 18853–18858

Matsumoto M, Seya T and Nagasawa S (1992) Polymorphism and proteolytic fragments of granulocyte membrane cofactor protein (MCP, CD46) of complement. *Biochem J* **281**: 493–499

*© 1999 Cancer Research Campaign*

Matsumura N, Tagami H, Hotta T, Takemura S, Yoshikawa T and Kondo M (1981) Serum complement profile and its clinical significance in patients with hepatocellular carcinoma and liver cirrhosis. *Nippon Shokakibyo Gakkai Zassi* **78**: 1753–1759

McNearney T, Ballard L, Seya T and Atkinson JP (1989) Membrane cofactor protein of complement is present on human fibroblast, epithelial and endothelial cells. *J Clin Invest* **84**: 538–545

Nagura H, Hasegawa H, Yoshimura S and Watanabe K (1985) The third (C3) and fourth (C4) components of complement in human liver. Immunocytochemical evidence for hepatocytes as the site of synthesis. *Acta Pathol Jpn* **35**: 71–78

Niehans GA, Cherwitz DL, Staley NA, Knapp DJ and Dalmasso AP (1996) Human carcinomas variably express the complement inhibitory proteins CD46 (membrane cofactor protein), CD55 (decay accelerating factor), and CD59 (protectin). *Am J Pathol* **149**: 129–142

Scoazec J-Y, Delautier D, Moreau A, Durand F, Degott C, Benhamou J-P, Belghiti J and Feldmann G (1994) Expression of complement-regulatory proteins in normal and UW-preserved human liver. *Gastroenterology* **107**: 505–516

Seya T and Atkinson JP (1989) Functional properties of membrane cofactor protein of complement. *Biochem J* **264**: 581–588

Seya T, Hara T, Matsumoto M, Sugita Y and Akedo H (1990) Complement-mediated tumor cell damage induced by antibodies against membrane cofactor protein (MCP, CD46). *J Exp Med* **172**: 1673–1680

Seya T, Matsumoto M, Hara T, Hatanaka M, Masaoka T and Akedo H (1994) Distribution of C3-step regulatory proteins of the complement system, CD35 (CR1), CD46 (MCP), and CD55 (DAF), in hematological malignancies. *Leuk Lymphoma* **12**: 395–400

Simpson KL, Houlihan JM and Holmes CH (1993) Complement regulatory proteins in early human fetal life: CD59, membrane cofactor protein (MCP) and decay-accelerating factor (DAF) are differentially expressed in the developing liver. *Immunology* **80**: 183–190

Simpson KL, Jones A, Norman S and Holmes CH (1997) Expression of the complement regulatory proteins decay accelerating factor (DAF, CD55), membrane cofactor protein (MCP, CD46) and CD59 in the normal human uterine cervix and in premalignant and malignant cervical disease. *Am J Pathol* **151**: 1455–1467

Theodore W, Post M, Liszewsky K, Anams EM, Tedja I, Miller A and Atkinson JP (1991) Membrane cofactor protein of the complement system: alternative splicing of serine/threonine/proline-rich exons and cytoplasmic tails produces multiple isoforms that correlate with protein phenotype. *J Exp Med* **174**: 93–102

*© 1999 Cancer Research Campaign* 
